# Density-Dependent Growth in Invasive Lionfish (*Pterois volitans*)

**DOI:** 10.1371/journal.pone.0066995

**Published:** 2013-06-25

**Authors:** Cassandra E. Benkwitt

**Affiliations:** 1 Department of Zoology, Oregon State University, Corvallis, Oregon, United States of America; Biodiversity Insitute of Ontario - University of Guelph, Canada

## Abstract

Direct demographic density dependence is necessary for population regulation and is a central concept in ecology, yet has not been studied in many invasive species, including any invasive marine fish. The red lionfish (*Pterois volitans*) is an invasive predatory marine fish that is undergoing exponential population growth throughout the tropical western Atlantic. Invasive lionfish threaten coral-reef ecosystems, but there is currently no evidence of any natural population control. Therefore, a manipulative field experiment was conducted to test for density dependence in lionfish. Juvenile lionfish densities were adjusted on small reefs and several demographic rates (growth, recruitment, immigration, and loss) were measured throughout an 8-week period. Invasive lionfish exhibited direct density dependence in individual growth rates, as lionfish grew slower at higher densities throughout the study. Individual growth in length declined linearly with increasing lionfish density, while growth in mass declined exponentially with increasing density. There was no evidence, however, for density dependence in recruitment, immigration, or loss (mortality plus emigration) of invasive lionfish. The observed density-dependent growth rates may have implications for which native species are susceptible to lionfish predation, as the size and type of prey that lionfish consume is directly related to their body size. The absence of density-dependent loss, however, contrasts with many native coral-reef fish species and suggests that for the foreseeable future manual removals may be the only effective local control of this invasion.

## Introduction

Invasive species are a major driver of biodiversity loss and can cause extensive ecological and economic impacts [1]. Invasive species often reach high population abundances which, combined with strong individual effects, drive their detrimental effects on native ecosystems [2]. Therefore, understanding what drives and regulates population dynamics of invasive species is crucial to predict and manage invasions effectively.

Direct demographic density dependence, which occurs when per capita gain rates decrease and/or loss rates increase as population size increases, is necessary for population regulation [3]. Density dependence can be manifested in the loss rates of mortality and emigration, and the gain rates of immigration, birth, and related rates of fecundity and individual growth (review by [4]). When a population is regulated, direct density dependence leads to a positive population growth rate at low densities and a negative population growth rate at high densities.

Although direct density dependence is a central concept in ecology [3], [4], it is not well-studied in most invasive species. Previous studies of density dependence in invasive species have often focused on the Allee effect (inverse density dependence) and its role in their establishment and population dynamics [5]. Studies which have investigated the role of direct density dependence in limiting invasive populations are usually observational field studies (e.g., [6]–[8]) or laboratory experiments (e.g., [9], [10]). Manipulative field experiments, which offer the most powerful tests for density dependence, are absent from the invasive animal literature. While difficult to conduct, manipulative field studies can provide novel insights into population regulation of invasive species by elucidating causal mechanisms of density dependence in the environment. Here, to my knowledge, I provide the first experimental field test of density dependence in any invasive animal and the first evidence of any kind for density dependence in an invasive marine fish.

The Indo-Pacific red lionfish (*Pterois volitans*) is an invasive marine predator that currently threatens reef ecosystems throughout the Western Atlantic, Caribbean, and Gulf of Mexico [11]. Since the early 2000s, lionfish populations have been spreading rapidly and increasing exponentially [12]. Lionfish densities in their invaded range exceed those in their native range by orders of magnitude [13], with the highest densities of >390 individuals per hectare recorded in the Bahamas [14]. Invasive lionfish are highly effective predators, as they over-consume a broad range of native coral-reef fishes, including economically and ecologically important species such as grouper and parrotfishes [15]–[17]. Due to their hitherto unchecked population explosion and their strongly negative effects on native coral-reef species, lionfish were recently listed as one of the world’s top conservation concerns [18]. Thus far, there is no evidence of a natural population control, so determining whether and at what population threshold density dependence will begin to limit lionfish populations is an essential step to understanding this invasion. Determining the role of density dependence in invasive lionfish may also inform management by projecting the trajectory of their populations and the effectiveness of removal strategies.

To test for evidence of density dependence in invasive lionfish, I conducted a manipulative field experiment in which I adjusted lionfish densities on small reefs to encompass a range of both naturally observed and artificially inflated densities. By tracking their individual growth, recruitment, immigration, and loss (mortality+emigration), I determined whether any of the measured lionfish demographic rates were density-dependent.

## Results

**Figure 1:**
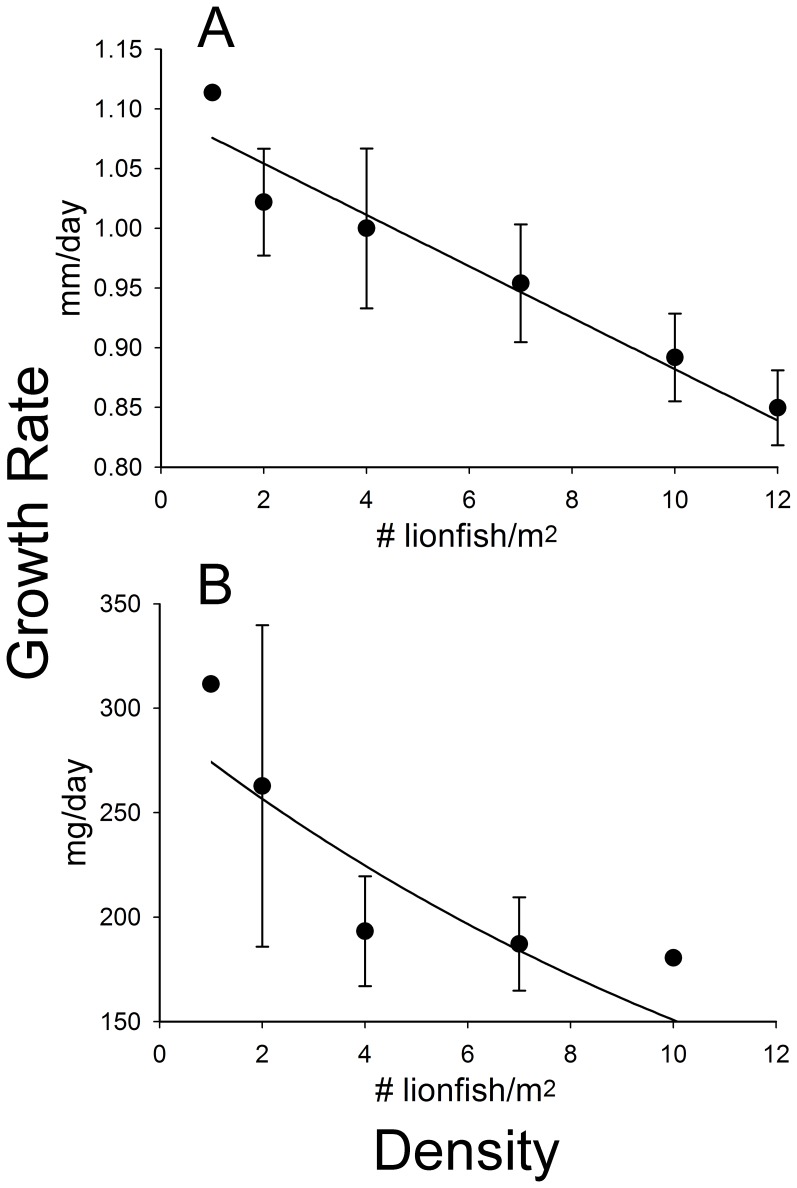
Effect of density on individual lionfish growth rates. Invasive lionfish growth rate in (A) length (mm/day) decreased linearly with increasing density throughout the 8-week experiment, while lionfish growth rate in (B) mass (mg/day) decreased exponentially. Points represent mean ± SEM. Curves show fitted regression lines from (A) a linear regression and (B) a non-linear regression using an exponential decline function. Sample sizes (# lionfish): (A) n for each point is: 1, 2, 4, 7, 10, and 12, respectively. (B) n for each point is: 1, 2, 3, 6, and 1, respectively.

Invasive lionfish exhibited density-dependent growth in length, with lionfish growing slower on reefs with higher lionfish densities (linear mixed effects model: χ^2^ = 18.72, df = 34, *P*<0.001). When averaged over the entire experiment, growth rate decreased linearly with increasing lionfish density (linear regression: t = −3.56, df = 34, *P = *0.001, Figure 1A). For each additional lionfish on a reef, lionfish grew 0.02 mm/day slower (95% CI 0.01 to 0.03). Lionfish growth in length was also affected by time, with lionfish growing slower on all reefs as the experiment progressed (linear mixed effects model: χ^2^ = 15.24, df = 86, *P*<0.001). There was no significant interaction between density and time (linear mixed effects model: χ^2^ = 1.95, df = 86, *P* = 0.162), however, indicating that lionfish growth rates over the course of the experiment were not differentially affected by density. Thus, lionfish across all density treatments exhibited similar declines in growth rate over time, but at higher densities they experienced slower growth rates than at lower densities throughout the entire experiment.

Lionfish growth in mass was also density-dependent (Figure 1B), but unlike length did not decrease linearly with increasing lionfish density. Instead, lionfish growth in mass was modeled by the negative exponential equation:
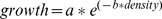
where *growth* is in mg/day, *a* and *b* are constants, and *density* is in #/m^2^. According to the non-linear regression model, both *a* and *b* were significant (non-linear regression: t = 6.59, df = 11, *P*<0.001 and t = 2.24, df = 11, *P* = 0.047, respectively) and were estimated to be 293.13+/−44.50 and 0.066+/−0.030, respectively.

There was no evidence of density dependence in lionfish loss rate (quasi-binomial regression: t = −2.04, df = 4, *P = *0.111). Loss rates were low across all density treatments, with only 6 out of 40 lionfish lost, about half of which occurred during the first 2 weeks of the experiment. At least one lost lionfish was due to emigration, as this tagged fish was found on an adjacent reef 200 m away. Despite thorough searches of surrounding areas, no other lost lionfish were found.

**Figure 2:**
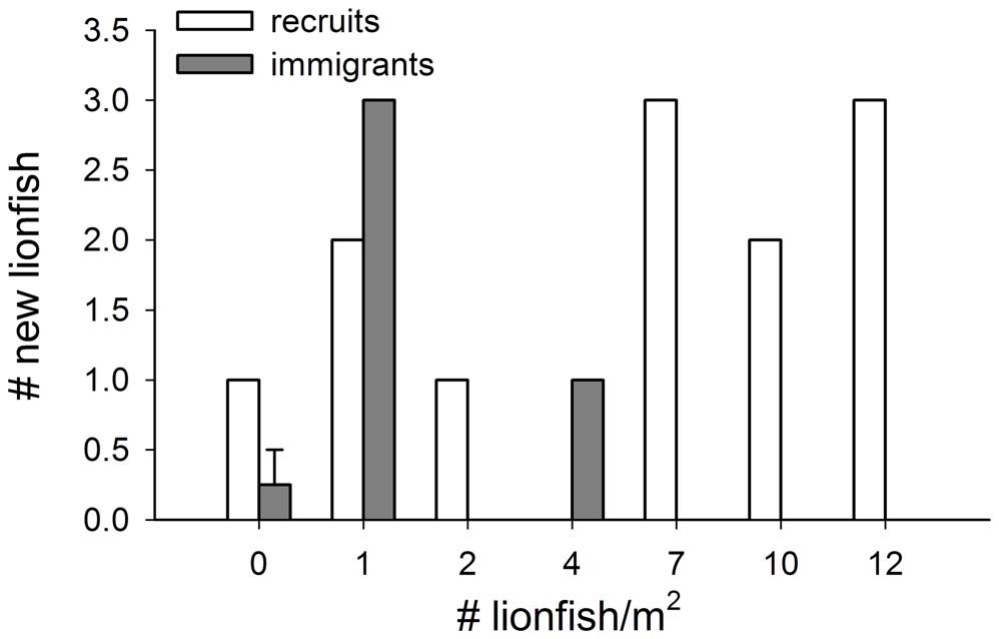
Number of new invasive lionfish recruits and immigrants by current lionfish density on each reef. Recruits are new lionfish ≤50 mm TL and immigrants are juvenile/adult lionfish >50 mm TL. Bars represent total number ± SEM if applicable. Sample sizes (# reefs): 4 for the 0 lionfish density treatment and 1 for all other densities.

There was also no evidence of density dependence in lionfish larval recruitment and juvenile/adult immigration (Poisson regression: z = 1.59, df = 8, *P* = 0.112 and negative binomial regression: z = −0.35, df = 8, *P* = 0.729, respectively; Figure 2). A total of 14 lionfish recruits appeared during the experiment, with 1–3 fish recruiting to all but one reef. There were only 5 juvenile and adult immigrants, all of which appeared on reefs with lionfish densities ≤50% maximum natural density.

## Discussion

The observed density-dependent growth rates may affect invasive lionfish population sizes and their impacts on native species. Slower juvenile growth may limit population size because fecundity, and often survival, are directly correlated with body size in fishes [19]–[21]. Variations in lionfish growth rate will also affect which native species are susceptible to lionfish predation. Lionfish diets switch from mostly crustaceans to fishes as they grow [16], so slower lionfish growth rates may increase the total consumption of crustaceans. In addition, the size of prey that lionfish consume is directly correlated with their body size [16], with lionfish consuming prey up to 2/3 their body length [15]. Furthermore, because increased lionfish density led to slower growth within a matter of weeks, the effects of increased lionfish density on growth rates, and consequently on their impacts on native species, may be realized even over short time scales.

The most likely mechanism for density-dependent growth in invasive lionfish is intraspecific competition. Within-species interference and exploitative competition for food and/or space causes density-dependent growth in a variety of fish species [19] including some invasive freshwater fishes [8], [22], [23]. Exploitative competition for food, rather than interference competition, was more likely in this case because no aggression between lionfish was observed during this experiment. Recruitment of native fishes also declined with increasing lionfish density throughout this study, providing further evidence for exploitative competition for food. Because lionfish are extremely effective predators [15]–[17], the consequence of within-species competition for food is that the invasion may eventually be controlled by lionfish over-consuming native prey, which would be a worst case scenario [12]. There is already some evidence that this may be the case, as lionfish abundance began stabilizing as prey fish declined on some Bahamian coral reefs, though it is difficult to disentangle the potential role of culling efforts and natural density-dependent processes [17].

Although invasive lionfish exhibited density-dependent growth, loss rates were comparable across all density treatments. Therefore, there was no indication of a population threshold at which lionfish will begin to decline, even though lionfish were experimentally bolstered to 150% natural observed densities. This finding contrasts with many native coral-reef fish species in which density-dependent mortality has been found using similar experimental methods in the same region [24]–[26]. However, unlike many native species, lionfish are not subject to high predation, most likely due to their venomous spines and other defensive characteristics [27]. Thus far, the only published report of predation on lionfish in their invaded range was by large grouper [28], which were present on my study reefs. Even grouper, however, do not appear to be common predators of lionfish, as a manipulative field experiment conducted on similar patch reefs demonstrated that Nassau grouper abundance does not affect lionfish loss rates (TJ Pusack, unpublished data). In addition to escaping native predators, lionfish appear to be free from other causes of density-dependent mortality, including interspecific competition [29] and parasitism (LJ Tuttle, unpublished data).

Since lionfish appear to be largely free from common sources of density-dependent mortality, it is likely that the low level of lionfish loss during this experiment was due to emigration. This study was conducted on juvenile lionfish inhabiting isolated patches, so the observed loss rates may not be comparable to lionfish in continuous habitats or larger lionfish. While the experimental patches were comparable in size to natural patch reefs found throughout the Bahamas, it is plausible that lionfish in more continuous habitats may exhibit more movement. However, in at least one estuarine system, juvenile and young adult lionfish up to 256 mm TL in a continuous habitat exhibited extremely high site fidelity [30]. Furthermore, studies of density-dependent loss in native coral-reef fishes in the same region have shown that small-scale results accurately scale up to larger habitats [25], [31], [32]. Along with habitat, lionfish size may be another important factor affecting emigration. Preliminary tagging studies indicate that smaller lionfish exhibit stronger site fidelity than adult lionfish in the Bahamas (personal observation), so future studies should investigate whether local adult lionfish density influences emigration rates.

As with loss rates, gain rates in terms of both recruitment of small lionfish and immigration of larger lionfish were similar across treatments. Because of the small number of lionfish that recruited and immigrated to all reefs throughout the experiment, there was low power to detect significant differences among treatments. Still, the observation that no lionfish immigrated to reefs with >50% natural maximum lionfish density may indicate an important area of future research. Given that the current method of lionfish management is through manual removal, if compensatory recruitment and/or immigration occur at sites with lionfish removal efforts, then culling of lionfish must be maintained at regular intervals [33], [34]. However, it is important to note that removals typically focus on adult lionfish, therefore conducting a study of the effects of adult lionfish density on population gain rates would be useful in informing management efforts.

Although this study was conducted on juvenile lionfish inhabiting isolated patch reefs, the findings may nonetheless have implications for management of the lionfish invasion. Because reductions in juvenile growth can eventually translate into limited adult abundances [19]–[21], the observed density-dependent growth may eventually cause local population regulation. However, the link between juvenile growth and population regulation is presently tenuous, and loss rates of juvenile lionfish were not density-dependent, at least over time periods typical of other young reef fishes. Furthermore, because density dependence typically manifests during the juvenile stages of coral-reef fishes [26], [35], [36], it is unlikely that adult lionfish will experience density-dependent loss, at least until native prey are severely depleted. Thus, it will be difficult to accurately predict when invasive lionfish populations will naturally level-off, and current efforts to reduce local densities via manual removal by divers [33], [34] are likely to remain the most effective management strategy. At the same time, demographic rates of adult lionfish should be monitored to evaluate the existence of any compensatory density dependence that may hinder removal efforts.

## Materials and Methods

### Ethics Statement

This study was approved by Oregon State University’s Institutional Animal Care and Use Committee (Permit Number: ACUP ID 3886), and all fish were handled in strict accordance with their guidelines. To minimize suffering, at initial capture lionfish were held in flow-through aquaria for a limited amount of time. All tagging and measuring was done in less than one minute per fish, so did not require anesthetic. Subsequent re-measurements were all done *in situ* to avoid removing fish from the water. Permits to conduct this field study were obtained from the Bahamian Department of Marine Resources. No protected species were sampled.

### Study System

To test for density dependence in local populations of invasive lionfish, I conducted a manipulative field experiment during June - August 2011 at Lee Stocking Island, Bahamas. I used a matrix of artificial patch reefs that was constructed in 1991–1992 and is located on a shallow (<4 m deep) sand and seagrass bank [37]. Each reef is separated from its nearest neighbor by 200 m and from the nearest land or continuous reef by at least 1 km. Each reef measures approximately 1 cubic meter and consists of 48 standard 8×8×16-in concrete blocks oriented to provide 24 holes [38]. Thus, the matrix of reefs provided habitat replicates of identical size and shelter, ensuring accurate density calculations (number of fish per m^2^). Over the two decades since construction, the reefs have become essentially natural features, supporting benthic communities of sponges, corals, and seaweeds that cover all surfaces, as well as home sites for over 70 species of fish, with hundreds of individuals per reef. These reefs have been used successfully in a variety of other studies, including tests of density dependence in native fishes [38], [39] and the effects of invasive lionfish [15].

During initial surveys, a pair of divers conducted baseline censuses of the entire fish community on each reef, recording the abundance by body size (total length, TL) of each species. I removed any lionfish and native piscivores and standardized the number of any strong interactors (Nassau grouper, *Epinephelus striatus*, and territorial *Stegastes* damselfish species) to mean natural densities to reduce any confounding effects of variation in the abundance of these species [40]. Consequently, all reefs had similar relatively intact yet standardized fish communities at the start of the experiment.

### Experimental Design

I studied new recruit (≤50 mm TL) and juvenile (50–71 mm TL) lionfish because density dependence in coral-reef fishes typically occurs in these stages [26], [35], [36]. I collected lionfish from nearby reefs on scuba using handnets. Captured lionfish were held in 190-l flow-through aquaria prior to release onto the experimental matrix. I tagged all lionfish subcutaneously using colored elastomer (Northwest Marine Technology Inc., Shaw Island, Washington, USA) on the caudal peduncle and/or slightly anterior to the caudal peduncle just under the dorsal fins. Each fish was given a unique tag based on the color and location of the elastomer, enabling me to identify individuals throughout the experiment [41]. All fish were held for at least 12 hours after tagging to allow for recovery from any tagging effects. There was no mortality from tagging. All lionfish were measured (TL to the nearest 1 mm) and weighed (wet weight WW to the nearest 1 mg) just before being released onto the experimental reefs.

Between June 26 and July 7, 2011, I transplanted lionfish onto 6 reefs at 6 different densities. I also established and maintained 4 reefs with 0 lionfish as controls. All treatments were started within a 2-week period. The treatment densities encompassed a range of both natural and artificially inflated densities. The highest maximum natural lionfish density observed on the reefs was 8 lionfish/m^2^, and the highest treatment density was 12 lionfish/m^2^ (150% maximum natural density). Since initial community assemblages among reefs were similar, lionfish density treatments were assigned via constrained randomization to ensure that similar densities were not clustered spatially. On each reef, all lionfish were released at the same time to avoid any potential priority effects [40]. To account for any losses due to transplantation, I used the number of lionfish present on each reef after 24 hours as the initial treatment density.

### Lionfish Loss

I recorded the number and identity of tagged lionfish present on each reef weekly. If a lionfish was not seen on a reef, I searched the surrounding sand and seagrass for approximately 10 minutes. If the lionfish was still not found, it was marked as absent for that week. If never found again, it was marked as lost from the last day it was seen. Total lionfish loss was calculated as the difference between the initial treatment density and the density on the final day of the experiment. To determine the effect of initial lionfish density on lionfish loss, I used a quasi-binomial regression (*R* version 2.14.2). Lionfish loss was minimal, yet to account for changes during the course of the experiment I calculated the weighted average weekly lionfish density for each reef. I used these weighted densities (1, 2, 4, 7, 10, and 12 lionfish/m^2^ rounded to the nearest fish) in all other analyses.

### Lionfish Gain

I recorded the number of new lionfish recruits (≤50 mm TL) and juvenile/adult immigrants (>50 mm TL) present on each reef weekly. Any new lionfish were immediately removed to preserve the treatment densities. To determine the effect of lionfish density on the total number of new lionfish recruits and immigrants on each reef, I ran Poisson regressions. Because the data were overdispersed in the regression with immigrants, I also ran a negative binomial regression. The results were similar between the two analyses, so I report the results from the negative binomial regression since diagnostic plots showed that this model better met the assumptions of the test (*R* version 2.14.2 with associated package *MASS*).

### Lionfish Growth in Length

Initial lionfish lengths ranged from 40 to 71 mm TL, and there was no significant difference in initial lengths among experimental treatments (ANOVA, F_1,34_ = 0.705, p = 0.407). Every two weeks, I recaptured all tagged lionfish on scuba using handnets, re-measured them *in situ*, and immediately released them back to their original locations on the reef. To account for repeated measures of individual lionfish over the course of the experiment, I ran a linear mixed effects model with lionfish density and time as fixed effects and individual lionfish as a random effect. Comparisons of models with and without a correlation structure using AIC values revealed that that no correlation structure was necessary. Because my sample design was unbalanced and because there was no significant interaction between density and time in the model, I used a type II sums of squares test to determine significance of my explanatory variables (*R* version 2.14.2, with associated packages *nlme*, *lme4*, and *car*).

I also used a simple linear regression to determine the effect of lionfish density on growth rate in length averaged over the entire experiment (*R* version 2.14.2). While this analysis does not provide information on growth rates over time, it provides a useful comparison to the effect of density on mass (see below) for which I only have measurements at the beginning and end of the experiment. Visual analysis of residual plots and formal tests (Shapiro and Levene’s) revealed that the assumptions of normality and homoscedasticity were met.

### Lionfish Growth in Mass

Initial lionfish masses ranged from 406 to 3147 mg WW. There was no significant difference in initial masses among treatments (ANOVA, F_1,34_ = 1.5, p = 0.229). After 8-weeks, lionfish were re-captured and re-weighed. However, Hurricane Irene, which passed from August 23 to 26, 2011, precluded re-weighing all lionfish but one from the 10-lionfish treatment and all lionfish from the 12-lionfish treatment. Therefore, analyses on lionfish growth rates in mass excluded the majority of the 10-lionfish treatment and all of the 12-lionfish treatment. Because there appeared to be a non-linear relationship between lionfish density and growth rate in mass, I compared a simple linear regression and a non-linear regression using a negative exponential formula using AIC values (*R* version 2.14.2). I report the results using the model with the lowest AIC value.
